# Coarse-Grained Molecular Dynamics Study of the Melting Dynamics in Long Alkanes

**DOI:** 10.3390/polym17182500

**Published:** 2025-09-16

**Authors:** Dirk Grommes, Olaf Bruch, Wolfgang Imhof, Dirk Reith

**Affiliations:** 1 Institute of Technology, Resource and Energy-Efficient Engineering (TREE), Bonn-Rhein-Sieg University of Applied Sciences, Grantham-Allee 20, 53757 Sankt Augustin, Germany; olaf.bruch@h-brs.de (O.B.); dirk.reith@h-brs.de (D.R.); 2 Institute of Integrated Natural Sciences, University of Koblenz, Universitätsstraße 1, 56070 Koblenz, Germany; imhof@uni-koblenz.de; 3 Fraunhofer Institute for Algorithms and Scientific Computing (SCAI), Schloss Birlinghoven, 53754 Sankt Augustin, Germany

**Keywords:** mesoscale coarse-graining, alkane, crystal melting, premelting

## Abstract

The melting behavior of alkanes plays a critical role in a wide field of applications. While experimental studies have established the occurrence of premelting phenomena in both short- and long-chain alkanes, molecular-level insights remain limited. In this work, we employ coarse-grained molecular dynamics simulations to investigate the melting behavior of high-molecular-weight alkanes, with a particular focus on continuous premelting dynamics in the transition regime toward polymer-like systems. By simulating alkane chains of varying lengths and analyzing temperature-dependent structural changes, we identify a crossover from discrete phase transitions to a gradual premelting process beyond chain lengths of N≈40 coarse-grained beads. The extrapolation of melting temperatures to zero heating rate yields values that agree well with the experimental data for the longest simulated chains. Compared to previous simulation studies, the slower heating rates used here offer enhanced quantitative agreement. Overall, the results provide new molecular-level insights into the melting of long-chain alkanes and highlight the utility of coarse-grained models in capturing complex phase behavior.

## 1. Introduction

The study of the melting behavior of alkanes (C_n_H_2n+2_) is of significant scientific interest due to its relevance in fields such as materials science, petrochemistry, and molecular thermodynamics. Alkanes serve as fundamental components in various industrial applications, including lubrication, energy storage, and phase change materials [1,2,3]. Understanding their melting properties at the molecular level is crucial for optimizing these applications and for developing accurate thermodynamic models.

For short- and medium-length alkanes, experimental studies have demonstrated the occurrence of so-called rotator phases (RI up to RV) during the melting process [4,5,6,7]. In the literature, the formation of such phases is described as a form of premelting, as it is an intermediate phase between the fully ordered crystalline solid and the isotropic liquid state [8]. The rotator phases still represent a crystalline ordered structure. They remain stable only within a narrow temperature range of a few Kelvins [9]. In the RI phase, disorder arises due to the rotation of entire molecular chains through a random rotation of ±90 degrees around their long molecular axes. The phase transitions from a fully crystalline state to the rotator phases are first-order transitions [9]. Rotator phases have been observed in n-alkanes with chain lengths n≥9, beginning with the appearance of the RI phase [7,8]. With an increase in chain length up to n=43, the system exhibits a growing number of distinguishable rotator phases [6]. Exact mechanisms depend on whether the number of CH_2_ units *n* is even or odd, respectively [10].

For long-chain alkanes, different phenomena are observed, which can also be understood as premelting in a continuously evolving manner over time. In this case, the terminal groups of long-chain alkanes undergo non-concerted segmental motions in combination with lateral translations below the melting point [11]. This leads to disorder at the crystal surface as well as end-group rotations. In the present study, only the latter phenomenon is considered and investigated as premelting.

On the experimental side, numerous studies have examined the premelting behavior of alkanes. Among others, NMR [12,13], X-ray scattering [5,14], differential scanning calorimetry [15,16], and Raman [17] and infrared spectroscopy [18] are suitable techniques for resolving the microstructures involved.

In addition, molecular dynamics (MD) simulations provide a powerful tool for investigating the phase transitions of alkanes with atomic-scale resolution [19,20]. Unlike experimental techniques, which are often limited by their spatial and temporal resolution, MD simulations allow for the direct observation of microscopic melting mechanisms, including nucleation or molecular rearrangements. The melting behavior of short-chain alkane systems is observed in several studies [1,21,22]. The occurrence of rotator phases is the focus of [8,9,10,20]. With respect to the investigation of long-chain alkanes, the study of Hall et al. [23] is highly interesting. They use a coarse-grained MD model for the investigation of C_171_H_344_ systems for observations of crystal melting.

Despite the extensive experimental and theoretical studies on the phase transitions of hydrocarbons, many fundamental questions remain open, particularly regarding the molecular mechanisms governing the melting process. In this work, we employ coarse-grained molecular dynamics simulations to investigate the melting behavior of high-molecular-weight alkanes. Our aim is to elucidate the continuous premelting behavior in the transition region from alkanes to long polymers. We especially focus on the impact of the coarse-graining approach on the results. Our findings contribute to a deeper understanding of the microscopic processes underlying alkane-melting and provide a basis for improving theoretical models of phase transitions in molecular systems.

## 2. Simulation Methodology

### 2.1. Force Field and Equilibration

For reasons of consistency, the present study employs the coarse-grained polyethylene force field previously introduced and applied in [24,25,26]. Three consecutive CH_n_ units are represented by a single coarse-grained bead. A comprehensive description of the development and parameterization of this force field is provided in [27]. In the context of this study, it should be emphasized that the force field was originally developed for the simulation of alkanes. It is specifically optimized to reproduce thermophysical quantities, such as the experimental density and enthalpy of vaporization. For validation purposes, the model has been shown to accurately reproduce several experimentally measured properties of polyethylene, including the density of the amorphous phase at 293 K ($\rho_{\mathrm{amorph},293K}$), melt density ($\rho_{500K}$), coefficient of thermal expansion (CTE), glass transition temperature ($T_{g}$), and crystallization onset temperature ($T_{c,\mathrm{on}}$), as reported in [25].

**Figure 1:**
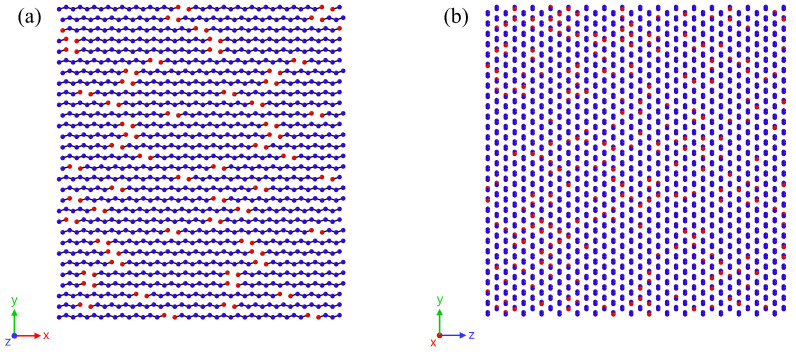
Structure of an initially generated system consisting of 2040 chains of chain length 20. For clarity, only a slice of the system is shown in (**a**). The orientation direction of chains is the x-axis. In (**b**), the hexagonal lattice structure can be identified. Particles marked in red represent the terminal ends of the chains.

The initial setup of the fully crystalline structures is performed in the following way: We construct one chain as a straight line, considering the equilibrium bond length and bond angle. Then, we place copies of this chain into the simulation box in a hexagonal grid. Grid spacing is chosen to closely match the minima of the Lennard–Jones potentials. It should be noted that, along the chain orientation direction, two chains are positioned sequentially within the simulation box. This arrangement prevents chains from interacting with each other due to periodic boundary conditions. Furthermore, the positions of the chain ends are statistically distributed within the box using a random algorithm. This procedure ensures that unintended local concentrations of chain ends are avoided. An example of a system generated in this manner is depicted in Figure 1. The number of chains arranged in the y- and z-directions is 30 and 34, respectively, for all systems.

The equilibration procedure is straightforward: we use the Berendsen thermostat ($\tau_{\text{thermo}}=1\;\mathrm{ps}$) and barostat ($\tau_{\text{baro}}=1000\;\mathrm{ps}$) to quickly (100 ps) heat up the system to 233.15 K at dt=4fs. Subsequently, the system is relaxed for 5 ns under unchanged conditions. This relatively short time-span is sufficient for a successful equilibration, as the systems were initialized in close proximity to their equilibrium state.

### 2.2. Simulation Procedure

The ESPResSo++ package (version 1.9.4.1) [28,29] was used to perform the molecular dynamics simulations. Starting with the equilibrated systems at 233.15 K, the systems were heated in the following way: different heating rates ranging from 0.625 K/ns to 4 K/ns were employed to induce melting in the systems. These values correspond to the overall duration of the heating process, ranging from 66.71 ns to 426.96 ns.

Temperature and pressure (ambient pressure of 1 bar) were controlled using a Berendsen thermostat ($\tau_{\text{thermo}}=1\;\mathrm{ps}$) and barostat ($\tau_{\text{baro}}=1000\;\mathrm{ps}$), respectively. We are aware that the Berendsen thermostat and barostat are not the ideal methods for controlling temperature and pressure in simulations aimed at determining melting temperatures. Nevertheless, other publications demonstrate that employing these methods can yield reliable results [22,23]. In particular, the study by Hall et al. [23], which we specifically employed for comparison with our results, applies Berendsen’s algorithms. The chain lengths *N* utilized are 20, 40, 60, 80, 100, and 120. Each system consisted of M=2040 chains. Corresponding initial system sizes ranged from 13.9×15.4×15.1 nm (N=20) to 81.5×15.4×15.1 nm (N=120). The heating process was terminated once the system reaches a temperature of 500 K.

### 2.3. Evaluation of the Microscopic Structure

To quantify the orientation of bond and chain end-to-end vectors, we referred to a well-established approach, as described in prior studies [30,31,32,33]. The orientation factor $\delta_{\mathrm{bond}}$ is defined in Equation (1) as the ensemble-averaged squared projection of the unit bond vectors ${\vec{e}}_{i}$, which connect successive beads along a polymer chain in a specified reference direction ${\vec{e}}_{\mathrm{testing}}$. The vector ${\vec{e}}_{\mathrm{testing}}$ was selected to align with the direction of interest, such as the principal or transverse deformation axis. The angular brackets denote an average over all bond vectors within the system:

(1) $$\delta_{bond}=\frac{3}{2}\langle {\left({\vec{e}}_{i}\;\cdot \;{\vec{e}}_{\mathrm{testing}}\right)}^{2}\rangle -\frac{1}{2}$$

The orientation factor for chain end-to-end vectors, $\delta_{\text{end-to-end}}$, was computed analogously, where ${\vec{e}}_{i}$ in Equation (1) is replaced by the normalized end-to-end vector ${\vec{e}}_{\text{end-to-end}}$ for each chain.

To further characterize the local orientational order of chain segments, the nematic order parameter *S* was employed, which is derived from the nematic tensor:

(2) $$Q_{\alpha \beta }=\frac{1}{N_{p}}\sum_{i=1}^{N_{p}}\left(\frac{3}{2}e_{i\alpha }e_{i\beta }-\frac{1}{2}\delta_{\alpha \beta }\right)$$

Here, $N_{p}$ denotes the number of evaluated bonds, and α,β∈x,y,z are Cartesian components. The global nematic order parameter *S* is defined as the largest eigenvalue of the tensor $Q_{\alpha \beta }$. The local nematic order parameter $S_{\mathrm{local}}$ is calculated for each bead *i* by including all bond vectors within a radial cutoff distance of $r_{\mathrm{cut}}=2\sigma$. The system-wide average, denoted as ${\bar{S}}_{\mathrm{local}}$, is obtained by averaging $S_{\mathrm{local}}$ over all beads. These calculations were carried out using the Freud analysis toolkit [34].

The degree of crystallinity in the system was determined based on the microscopic structural criterion outlined in [25]. This criterion considers both spatial proximity and orientational alignment between non-bonded beads. The key steps are as follows: (1) A considered bead must have another bead located in its vicinity at a shorter distance than $0.975\;\cdot \;2^{1/6}\sigma$. The first bonded neighboring beads are excluded here. (2) For beads that are positioned in close proximity according to criterion (1), the corresponding bond vectors must also exhibit a distinctly parallel orientation with respect to one another. (3) If three or more consecutive beads ($n_{\mathrm{stem}}\geq 3$) within a chain satisfy the defined geometric and orientational thresholds (cf. [25]), they are classified as crystalline.

The developed algorithm can further be applied to estimate the characteristic size of crystalline domains within a chain. For this purpose, the number of consecutive beads along a chain that satisfy criteria (1) and (2) is determined. According to our criterion, this number is defined as the crystal stem length $n_{\mathrm{stem}}$. Applying this procedure to all beads in the system allows the systematic quantification of the distribution of crystalline regions of varying lengths.

Chain entanglements are quantified by means of primitive path analysis (PPA), as originally proposed by Everaers et al. [35]. Within this approach, chains are contracted between fixed endpoints, while chain crossings are prevented by non-bonded interactions. Based on the tube model [36,37,38], the length of the resulting primitive path (PP) can be determined as $\langle L_{\mathrm{pp}}\rangle =\left(N-1\right)\langle b_{\mathrm{pp}}\rangle$, where $\langle b_{\mathrm{pp}}\rangle$ denotes the PP bond length. Furthermore, by calculating the mean-squared end-to-end distance $\langle R_{\mathrm{ee}}^{2}\rangle$, the entanglement length can be obtained as $N_{e}=\left(N-1\right)\langle R_{\mathrm{ee}}^{2}\rangle /{\langle L_{\mathrm{pp}}\rangle }^{2}$. Comprehensive details regarding the application of the PPA to systems analogous to those investigated in this work are provided in [25].

## 3. Results

In the following subsections, the effects during the melting process are described. The evaluation of results begins with a discussion of the chain length and heating rate dependent behavior. Subsequently, a microscopic analysis of different melting dynamics is presented.

### 3.1. Determination of the Crystal Melt Temperature

**Figure 2:**
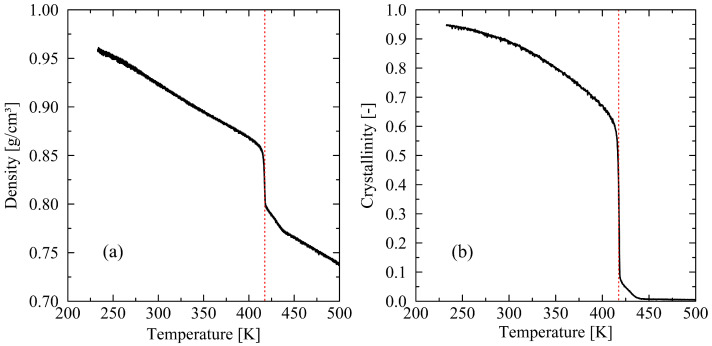
Plot of the density (**a**) and crystallinity (**b**) over temperature for chains with chain length N=120 and a heating rate of 0.625 K/ns. The dotted red line marks the crystal melt temperature, determined at the inflection point of density–temperature curve. For the results shown, the melting temperature of the crystal is 417.5 K.

First, we examine the general course of the melting process for systems composed of long chains (N=120). To this end, a representation of the density and the degree of crystallization as a function of temperature is particularly suitable. Both quantities provide insight into the temperature at which melting is expected to occur. As shown in Figure 2, the curve progression exhibits the characteristic spontaneous decrease in both quantities at the moment of crystal melting. The crystallite melting temperature is determined based on the inflection point in the density–temperature curve. This approach is consistent with the method employed by Hall et al. [23].

**Figure 3:**
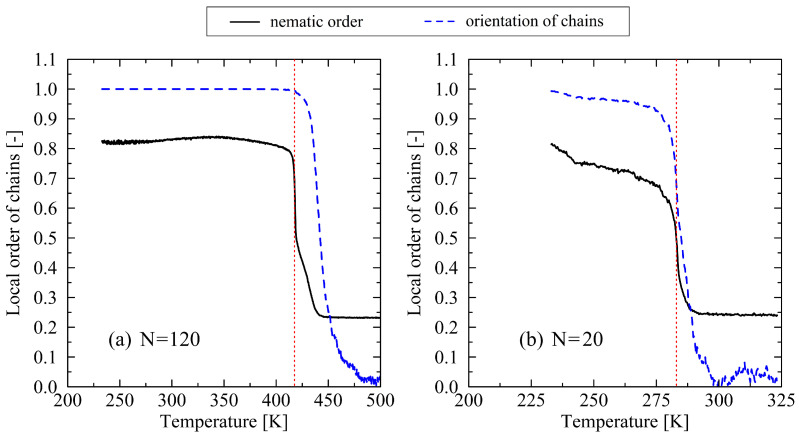
The nematic order parameter and orientation factor of the chain end-to-end vectors plotted over temperature. The figure contains the results for chain lengths N=120 (**a**) and 20 (**b**) at a heating rate of 0.625 K/ns. The dotted red lines mark the crystal melt temperatures according to the inflection point of the density–temperature curve. Please note that the x-axes in (**a**,**b**) are scaled differently.

To analyze the processes involved in the melting of crystalline structures in greater depth, it is useful to consider the local orientation of chain segments. Figure 3 illustrates the nematic order parameter, as well as the orientation factor of the chain ends. Both quantities exhibit analogous behavior to the progression of density and the degree of crystallinity. Notably, the value of the nematic order parameter spontaneously drops at the moment of crystal-melting. This behavior is also observed for the orientation factor of the chain ends. In cases of long chain length (Figure 3a), it is particularly noteworthy that the loss of orientation occurs gradually at first and exhibits a pronounced increase at temperatures several Kelvins above the melting point. Moreover, the orientation factor of the chain-end vectors remains nearly ideal, approaching a value of 1 up to the onset of melting. This indicates a complete retention of the original orientation of the chain ends prior to thermal disordering. The observed behavior suggests that the crystal melts from the interior rather than starting from the chain ends. In contrast, the behavior of very short chains (N=20), as shown in Figure 3b, deviates significantly. In this case, a de-orientation of the chain end vectors occurs even before the crystal has completely melted. Furthermore, the point at which the end-to-end vectors irreversibly lose their orientational alignment coincides almost exactly with the loss of nematic order.

**Figure 4:**
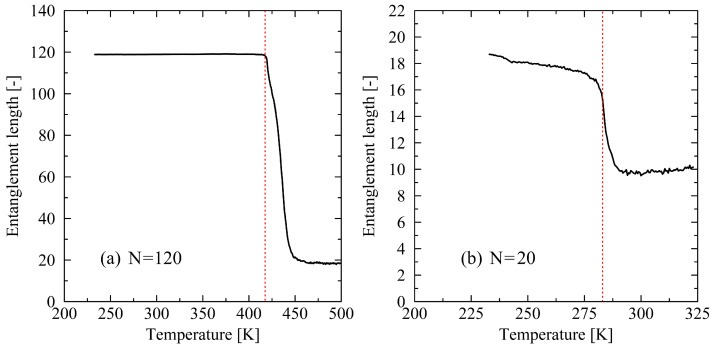
Representation of the entanglement length $N_{e}$ during the heating process for systems with chain lengths N=120 (**a**) and N=20 (**b**), obtained at a heating rate of 0.625 K/ns. The red dotted lines indicate the respective melting temperature of the system.

To obtain a deeper understanding of chain behavior, the entanglement length was additionally determined (Figure 4). In analogy with the order parameters describing the system (cf. Figure 3), chains of length N=120 exhibit stable behavior with respect to the entanglement length. An observed value close to Ne=120 indicates that the chains in the fully crystalline state are not entangled. Upon melting of the crystal, the entanglement length decreases sharply until a stable plateau is reached. This clearly indicates an entangling behavior of the chains toward a fully amorphous state. For the investigated chain length of N=20, a qualitatively similar trend is observed. However, in this case, deviations from the fully crystalline structure with respect to the entanglement length emerge at the onset of the heating process.

**Figure 5:**
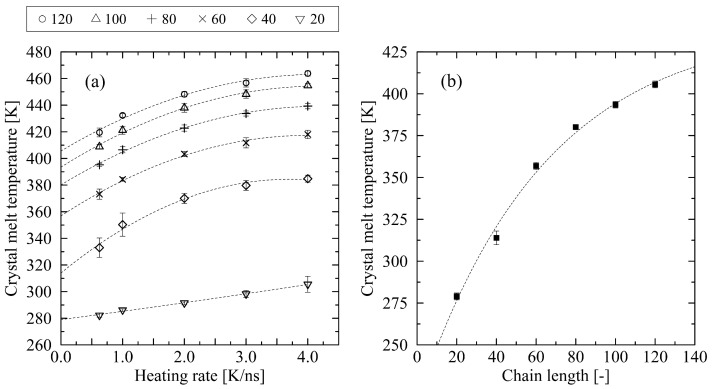
(**a**) Melt temperature plotted over different heating rates ranging from 0.625 K/ns to 4 K/ns. The figure includes all investigated chain lengths from N=20 to 120 CG beads. The dotted lines represent the second degree polynomial fits for each investigated chain length. In (**b**), the extrapolated data for a heating rate approaching zero are presented (see (**a**)). The dashed line shown here represents an exponential fit with limited growth ($f\left(x\right)=a-b\;\cdot \;e^{-cx}$, where *a*, *b*, *c* are the fitting parameters). Error bars are, in most cases, smaller than symbols.

If the objective is to determine a crystal’s melting temperature under comparable conditions to experimental settings, a heating rate that is as slow as possible must be employed. However, in molecular dynamics (MD) simulations, this approach is severely constrained by computational limitations. Therefore, an alternative method must be applied. A suitable approach involves determining the melting temperature for various heating rates and extrapolating the resulting trend to a heating rate approaching zero. Figure 5a presents the corresponding data, including the extrapolation, for different chain lengths. Additionally, Figure 5b shows the determined crystal melt temperatures for all chain lengths, including an exponential fit considering limited growth.

As demonstrated, the extrapolation of the data can be achieved using a second-degree polynomial fit. We determine a crystal melt temperature for N=100 at $T_{m,100}=393.4\pm 2.0$ K and for N=120 at $T_{m,120}=405.6\pm 2.1$ K, respectively. These results exhibit good agreement with the experimental findings, which are in the range between 403.6 K for C_294_H_590_ [39] and 408.15 K for C_378_H_758_ [40]. However, for shorter chain lengths, the simulation results deviate significantly from the experimental data. For example, at chain length N=60, the extrapolated crystal melt temperature $T_{m,60}$ is 356.8±2.1 K, which strongly differs from experimental values (399.15 K (C_159_H_320_) [41], 400.65 K (C_192_H_386_) [42]). The reasons for this inconsistent behavior are discussed in the following section, where the microdynamics of the melting process are analyzed.

### 3.2. Analysis of the Melting Dynamics

**Figure 6:**
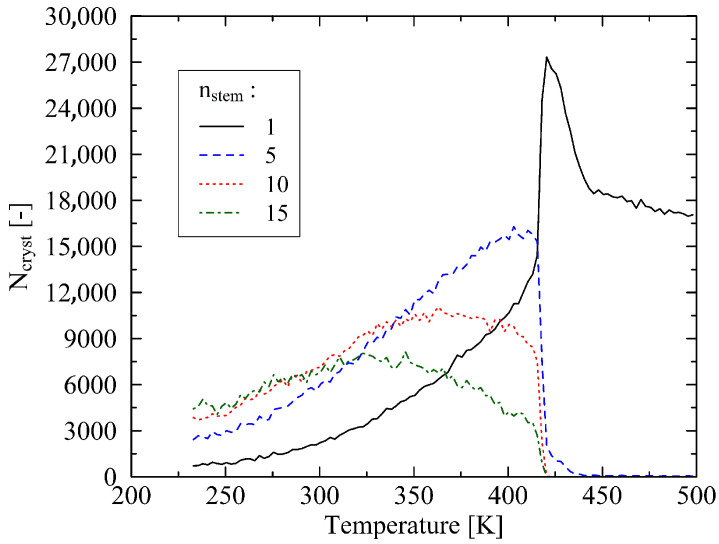
The figure illustrates the evolution of crystal melting behavior based on the stem length definition, as proposed by Grommes et al. [26]. The underlying system consists of polymer chains with a length of N=120, subjected to a heating rate of 0.625 K/ns. Exemplarily, the occurrence frequencies of stem lengths 1, 5, 10, and 15 within the system are depicted. $N_{\mathrm{cryst}}$ denotes the number of beads that belong to the respective stem length.

Initially, the dynamics of the melting process are analyzed by examining the stem length distribution, as described by Grommes et al. in [25]. In this context, Figure 6 provides insight into the number beads ($N_{\mathrm{cryst}}$) that belong to crystalline regions of a given length. The investigated system has a chain length of N=120. Using the example of inspected stem lengths of $n_{\mathrm{stem}}=1$, 5, 10, and 15, the data demonstrate a reduction in the length of crystalline domains as temperature increases. Correspondingly, the number of shorter crystalline regions initially increases until these, in turn, fragment into even shorter segments. This phenomenon explains the distinct peak observed in each distribution, which shifts toward higher temperatures for progressively shorter crystalline regions.

Notably, the protruding peak corresponding to $n_{\mathrm{stem}}=1$ is observed in all investigated systems. At the point of complete crystal melting, the structure remains close to crystalline density but is already locally disordered. This accounts for the observed behavior. It should be noted that the value of $n_{\mathrm{stem}}=1$ does not approach zero after complete melting. As discussed by Grommes et al. [25], this is attributed to the presence of randomly occurring, locally dense structures that, while not stably crystalline, persist in the system.

**Figure 7:**
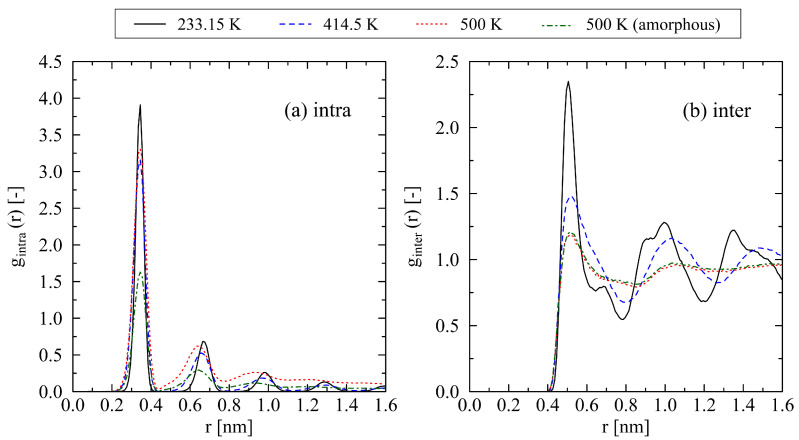
Intra-chain (**a**) and inter-chain (**b**) radial distribution functions for a system with chain length N=120 at different temperatures. The data were obtained using a heating rate of 0.625 K/ns. The temperature of 233.15 K corresponds to the onset of the heating process. At 414.5 K, the melting of the crystalline system starts, while at 500 K the system is in a fully amorphous state. For comparison, the results for an amorphous reference system (500 K (amorphous)) are included. This system was generated and equilibrated in a fully amorphous state according to the methods described in [24].

The analysis of the intra- and inter-chain radial distribution functions provides further insight into the system behavior. Results for a system with chain length N=120 are shown in Figure 7. The first peak in Figure 7a corresponds to the bond length. Up to a temperature of 414.5 K, the recurring peaks (corresponding to multiples of the bond length) indicate a highly ordered, crystalline structure of the chains. Only at 500 K does this behavior diminish for distances r>1 nm. The inter-chain radial distribution function (Figure 7b) reveals a similar trend. Up to 414.5 K, the ordered structure of the system remains clearly visible. At 500 K, the internal structure of the system nearly coincides with that of the fully amorphous reference system, which was generated and equilibrated according to [24].

**Figure 8:**
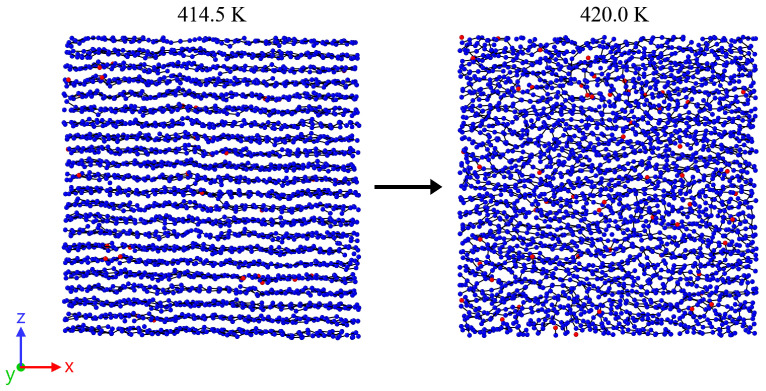
Representative snapshots for a system with a chain length N=120 and a heating rate of 0.625 K/ns. The dimensions of the cut-outs from the entire system are 10 nm × 10 nm (thickness 2.5 nm). At 414.5 K, the system is in a nearly ideal crystalline state, while at 420.0 K it is completely amorphous. The terminal groups of the polymer chains are highlighted in red. No local accumulation of chain ends is observed either immediately before or after the melting process. The crystal melts abruptly as a whole.

While the previously discussed behavior remains consistent across all investigated chain lengths, an explanation for the deviations in crystal melting temperature observed in Section 3.1 for short chain lengths still needs to be established. To this end, an inspection of the trajectory during the heating process is appropriate. This analysis reveals that, in cases with longer chain lengths, the crystal instantaneously melts as a whole (Figure 8). This specific behavior is indicated in Figure 3.

**Figure 9:**
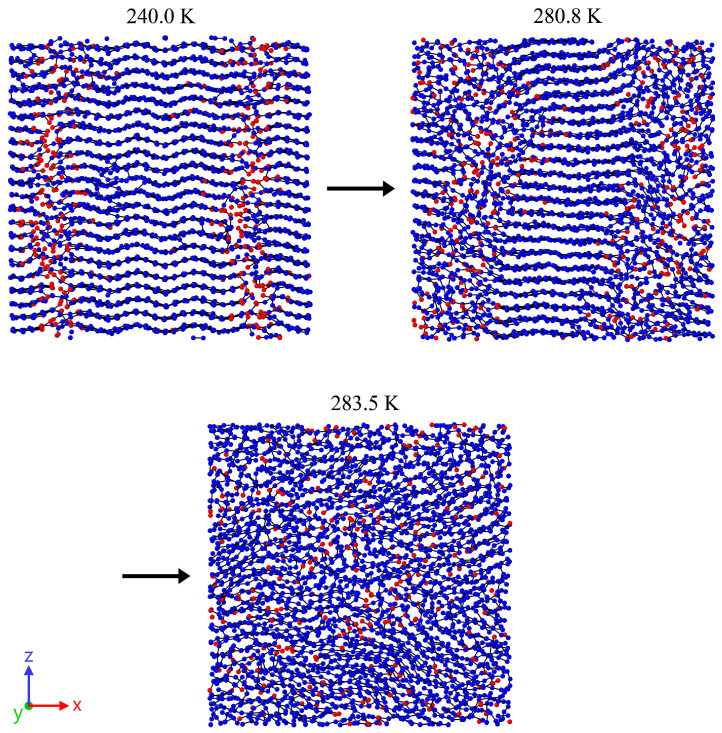
Representative snapshots for a system with chain length N=20 and a heating rate of 0.625 K/ns. The dimensions of the cut-outs from the entire system are 10 nm × 10 nm (thickness 2.5 nm). At 240.0 K, the system remains in a quasi-fully crystalline state; however, the chain end groups have already begun to locally aggregate (indicated by red markers). At 280.8 K, it is clearly observable that the crystal begins to melt, originating from the regions with concentrated end groups. At a temperature of 283.5 K, the system has transitioned into a fully amorphous state.

In contrast to longer chain lengths, short chains exhibit a distinctly different behavior. A concentration of chain ends during heating is monitored (Figure 9). This phenomenon can be attributed to the facile sliding of chains past one another.

**Figure 10:**
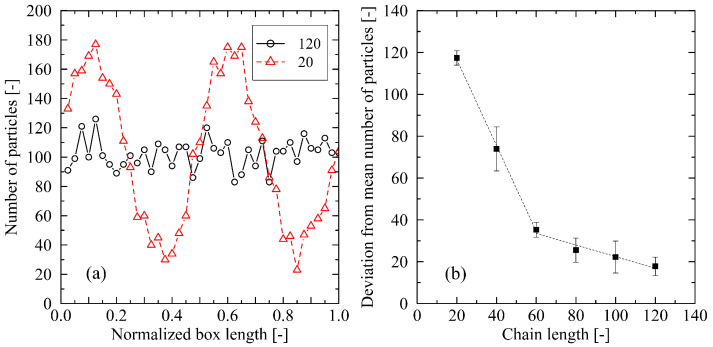
(**a**) Distribution of chain end groups along the direction of chain orientation. For the figure, the simulation box is divided into 40 evenly spaced segments. For each segment, the number of contained chain-end groups is determined. To ensure comparability, the data is presented with respect to the normalized box length. In (**b**), the average deviation in the number of chain ends per segment is calculated. Data are shown for all investigated chain lengths. The reported standard deviation refers to the six independently simulated systems for each chain length. All analyses are based on a cooling rate of 0.625 K/ns. Lines in (**a**,**b**) are a guide to the eye only.

Additionally, Figure 10a illustrates the observed behavior with respect to the spatial distribution of chain ends. For the investigated long chains (N=120), it can be shown that, immediately prior to crystal melting, the chain ends are uniformly distributed along the direction of chain orientation. In contrast, for shorter chains (N=20), distinct regions with a high concentration of chain ends are clearly visible.

The concentration of chain ends occurs within an extremely short time span and inherently represents a weakening or disruption of the crystalline structure. Consequently, the crystal begins to melt from the locally concentrated chain ends. Experimentally, this behavior is described as the so-called premelting phenomenon [11]. Studies have shown that this effect leads to a reduction in the melting temperature by approximately 30 K compared to unaffected regions. Figure 10b presents the averaged deviation in the number of chain end groups along the box length as a measure of the inhomogeneous distribution of chain ends. Each data point evaluates five snapshots of the trajectory (spaced at intervals of 200,000 time steps) at the moment of maximal inhomogeneity in the system, occurring shortly before melting. It becomes evident that a concentration of chain ends (as an indication of premelting) occurs immediately prior to melting for chain lengths of N=20 and 40. For chain lengths of N=60 and above, however, this effect becomes less pronounced.

Nevertheless, even when accounting for the premelting effect, the melting temperature of short chain systems is significantly underestimated. The discrepancies observed for the chain lengths of N=20 and 40 primarily stem from the mesoscale modeling approach. The use of a coarse-grained force field, which features significantly softer potentials compared to fully atomistic representations, shifts the system dynamics towards accelerated processes. In the present case, this results in the observed rapid sliding of chains past one another. For the chosen short chain length, this effect is disproportionately pronounced, leading to a considerable overestimation of the premelting behavior. This, in turn, results in a substantial underestimation of the crystalline melting temperature.

## 4. Discussion

Through the analysis of quantities such as local orientation, entanglement length, and the intra- and inter-chain radial distribution functions, the microscopic rearrangement processes governing the melting of crystalline structures are elucidated. Importantly, this analysis already demonstrates that short-chain systems (20≤N≤40) exhibit markedly different behavior from their long-chain counterparts.

Experiments demonstrate that the melting temperature of a crystalline material is influenced not only by the heating rate.

The cooling conditions during the crystallization process—particularly the crystallization temperature—also affect the morphology of the crystalline phase and the degree of crystallinity of the sample [40]. It can be generally stated that a heating rate of 1 K/min or lower is sufficiently slow to determine a thermodynamically meaningful melting temperature [42]. However, due to numerical limitations, the simulation operates on a timescale that does not permit cooling or heating rates as slow as those used in experiments. Nevertheless, by extrapolating the data to a zero heating rate, it is possible to derive values that are comparable to experimental observations. For the longest chain lengths studied, these extrapolated values are in good agreement with the experimental data. On the other hand, the results indicate that for even longer chains (N>120), a continued increase in the melting temperature is to be expected. A suitable exponential fit with asymptotic saturation yields an estimated ultimate melting temperature of 441.5±18.9 K. While this value is significantly higher than the experimental one, it remains acceptable within the scope of the simplified coarse-grained model used for polyethylene.

In comparison to other molecular dynamics simulations, particular reference must be made to the work of Hall et al. [23]. Their similar methodological approach, applied to a different force field, leads to results that deviate even further from the experimental values. Moreover, the slower cooling rate employed in the present study is a key factor enabling a more accurate extrapolation of the crystalline melting temperature.

Lastly, the simulation also reveals the premelting behavior. A distinct transition region is identified for chains with N≤40, beyond which premelting becomes the dominant mechanism in the melting dynamics.

In conclusion, the simulation qualitatively confirms the premelting effect observed in experiments. The underlying processes are reproduced in a highly illustrative manner.

## Data Availability

The data that support the findings of this study are available from the corresponding author upon reasonable request.
